# In the article “Website for families of non-breastfed children: development and validation of content and interface”

**DOI:** 10.1590/0034-7167.20257804e06

**Published:** 2025-09-01

Included:

