# Supplementary material for: In the article “Website for families of non-breastfed children: development and validation of content and interface”
Source: Rev Bras Enferm. 2025 Sep 1;78(4):e2025n4e06. doi: 10.1590/0034-7167.20257804e06 (PMC12407856; doi:10.1590/0034-7167.20257804e06)
Supplement: Related texts

# Correction: "Website para família de crianças não amamentadas: desenvolvimento e validação de conteúdo e de interface"

**DOI:** 10.1590/0034-7167.20257804e06pt

Included:
